# Use of double-stranded DNA mini-circles to characterize the covalent topoisomerase-DNA complex

**DOI:** 10.1038/srep13154

**Published:** 2015-08-24

**Authors:** Armêl Millet, François Strauss, Emmanuelle Delagoutte

**Affiliations:** 1 Structure et Instabilité des Génomes, Sorbonne Universités, Muséum national d’Histoire naturelle, Inserm U 1154, CNRS UMR 7196; CP26, 57 rue Cuvier 75005 Paris, France

## Abstract

The enzymatic DNA relaxation requires the DNA to be transiently nicked and rejoined, the covalent topoisomerase-DNA complex being a key intermediate of the nicking-joining reaction. Practically, this reaction is most often characterized by oligonucleotides. However, the incision-religation of an oligonucleotide does not fully recapitulate the incision-religation occuring during relaxation and the preferred substrate for such reaction characterization is supercoiled DNA. We therefore developed a method that used radiolabeled supercoiled DNA mini-circles to characterize the covalent enzyme-DNA complex formed during a relaxation reaction. Resolution of the relaxation products under different conditions permitted to quantify the proportion of covalent complex formed during the relaxation catalyzed by two topoisomerase models, the *Escherichia coli* topoisomerase I and the calf thymus topoisomerase I. As expected, the covalent complex formed with the calf thymus topoisomerase I was significantly enriched by camptothecin, a widely-used inhibitor of this topoisomerase, and a salt jump permitted the multiple topoisomerases trapped per mini-circle to complete the reaction cycle. The identified positions of the camptothecin-induced incision sites were shown to be independent of the linking number and the substrate circular nature Overall, our results demonstrate that supercoiled mini-circles constitute a powerful and polyvalent substrate to characterize the mechanism of action of novel topoisomerases and inhibitors, including the incision-religation reaction.

Topoisomerases are essential enzymes whose function is to regulate cellular DNA supercoiling and to solve topological problems associated with the manipulation of DNA (for reviews on DNA topoisomerases see^1^,^2^,^3^,^4^,^5^,^6^). The replication, transcription, repair, recombination or chromatin remodeling machineries indeed modify the DNA topology locally inducing positive or negative supercoils and create intermediates of complex topology, such as knots, catenanes or hemicatenanes that can become toxic if left unresolved. To modulate the topology of the DNA, topoisomerases start by nicking the sugar-phosphate backbone of the DNA, establishing a covalent bond with the DNA. From this nicked intermediate, either a strand passage takes place with a DNA strand passing through the break or a controlled rotation occurs with the rotation of the DNA duplex not covalently attached to the enzyme around the phosphodiester bond opposite the nick. At the end of these processes, the DNA backbone is resealed, the enzyme released, and the DNA global topology has been modified.

The classification of topoisomerases into families is based on the number of strands of the duplex DNA that are broken during the enzymatic reaction. Topoisomerases of family I break one strand of the duplex DNA whereas topoisomerases of family II introduce two breaks, one on each strand of the duplex DNA. Both families have been further divided into subfamilies (IA, IB, IC and IIA and IIB), depending on the polarity of the DNA end that is linked to the topoisomerase, the mechanism used to relax DNA, and the primary sequence of the protein. Topoisomerases of family IA, IIA and IIB change the topology by a strand passage mechanism whereas those of family IB and IC use a controlled rotation mechanism.

The covalent bond between the topoisomerase and the DNA is established between the lateral chain of the catalytic tyrosine in the protein and the 3′-phosphate (in the case of topoisomerases IB and IC) or the 5′-phosphate (in the case of topoisomerases IA and II) of the DNA. The resulting covalent complex is a short-lived species but its half-life time, which depends directly on the relative rates of the nicking-closing reactions, can be altered by drugs, such as camptothecin that decreases the joining rate^7^,^8^ or by protein partners of topoisomerases as suggested for the RecQ Mediated Instability 1 (RMI1) protein partner of the human topoisomerase III alpha^9^ and the yeast RMI1 homolog^10^. For the cell, the consequences of altering the relative rates of the nicking and joining reactions of a topoisomerase can be dramatic as exemplified by the cytotoxic effect of camptothecin or its derivatives (for reviews on the IB family of topoisomerase inhibitors see^11^,^12^).

Several types of substrates have been used to trap and characterize the covalent complex. The method based on plasmids relied on the fact that a nucleoprotein complex exhibited a lower buoyant density than free DNA when sedimented at equilibrium on an alkaline cesium chloride gradient^13^,^14^. However, the conditions of relaxation were not optimal for relaxation since they promoted the accumulation of nicked DNA instead of relaxed plasmids as final products^13^,^14^. Other pioneer assays using ss DNA of various lengths permitted to characterize the covalent complex formed with type IA topoisomerases^15^,^16^,^17^. Recently, procedures relying on the use of oligonucleotides have been developed to elucidate the mechanism of action of topoisomerase drugs and to facilitate comparison of the efficiency of different drugs in stabilizing the covalent complex^18^. Over the past decade, mutations of the *Yersinia pestis* and *Escherichia coli* Topoisomerase I have been isolated^19^, and the stabilization of the covalent complex by these point mutations has been inferred from the high level of cleaved oligonucleotides generated by these mutants^20^,^21^,^22^.

Whereas all these approaches using oligonucleotides are undoubtedly very valuable and have been very helpful in elucidating the mechanism of action of topoisomerases and their inhibitors, it is nevertheless worth noticing that the incision and religation of oligonucleotides do not fully recapitulate the nicking and closing reactions that occur during a relaxation reaction since no strand passage or controlled rotation takes place with such substrates. It may indeed well be that the relative kinetic of the incision and religation reactions be modulated by the strand passage step in the case of the topoisomerases of family IA and II or the swiveling step in the case of the topoisomerases of family IB. Our goal was therefore to develop a quantitative method that used the preferred and natural substrate of the nicking-joining reaction, i.e. supercoiled DNA, to characterize these two coupled steps of the topoisomerase-catalyzed relaxation reaction. Such method should also permit to estimate the proportion of covalent complex during a relaxation reaction, since topoisomerase mutations, topoisomerase protein partners and topoisomerase inhibitors may alter this proportion. As an alternative to plasmids, double-stranded DNA mini-circles (dsMCs) might represent the ideal relaxation substrate for such a study due first, to their small size permitting the resolution of all relaxation intermediates, including the covalent complex, and second, to the possibility of radiolabelling them, thus conferring a high detection sensitivity and a reliable quantification aspect to the method. We therefore prepared dsMCs of 235 base pairs (bps) and used them to characterize the covalent complex of two topoisomerase models, the *Escherichia coli* TopoI (*Ec* TopoI) of the IA family of topoisomerases and the calf thymus TopoI (Ct TopoI) of the IB family. Altogether, our results demonstrate that dsMCs are usable to resolve and isolate the covalent topoisomerase-DNA complex and therefore suitable to characterize the nicking-joining reaction of any DNA topoisomerase under various conditions (in the presence of protein partners or inhibitors, with mutant topoisomerases, etc.) Our method represents an improvement in the field of DNA topoisomerases since the coupled nicking-joining reaction is examined with the preferred and natural substrate of relaxation.

## Results

### Relaxation activity of the Ct TopoI and Ec TopoI on dsMCs

Seven topoisomers with relative linking numbers ranging from 0 to −6 (labeled in what follows T_i_ where i = relative linking number) were prepared (Supplementary Fig. 1 and Online Methods) and used to accurately assay the relaxation activity of two topoisomerase models, the Ct TopoI and the *Ec* TopoI (Supplementary Figs. 2, 3). The Ct TopoI and *Ec* TopoI efficiently relaxed the topoisomers with relative linking numbers ≤ −2. The final reaction products were T_0_ and T_+1_ for Ct TopoI and T_−1_ for *Ec* TopoI. The *Ec* TopoI had no activity on T_−1_ and T_0_ whereas the Ct TopoI had a strong and moderate activity on T_−1_ and T_0_, respectively.

### Visualization of a covalent complex during the relaxation of dsMCs by the Ct TopoI

The relaxation reaction involves the formation of a covalent topoisomerase-DNA complex. This intermediate is a short-lived species but during the incision of an oligonucleotide or longer ss DNA it can nevertheless be irreversibly trapped by adding SDS^15^,^16^. Since the incision of an oligonucleotide is not a perfect mimic of the incision reaction taking place during plasmid relaxation, we investigated whether the covalent complex could also be trapped during the relaxation of dsMCs. The relaxation of dsMCs by the Ct TopoI was then performed at 37 °C over a 10 min period, after which SDS was added to trap the covalent complex and stop the reaction. The sample was next split into two, with one half being treated with proteinase K, the other half not. Both samples were then analyzed by gel electrophoresis under several gel conditions.

**Figure 1:**
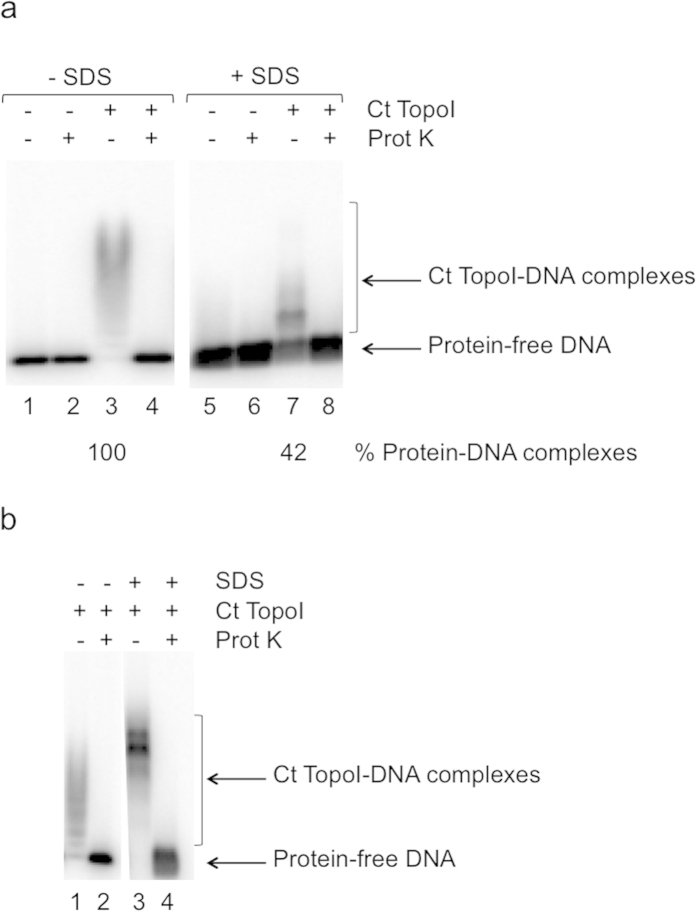
Relaxation of a mixture of the topoisomers T_−4_ and T_−5_ analyzed on an agarose gel under native conditions. (**a**) Relaxation was performed with the Ct TopoI at a concentration of 18.75 mU μL^−1^ (≈0.2 nM) as described in the methods section. After 10 minutes of incubation at 37 °C, SDS was added to the reaction mixtures to irreversibly trap the Ct TopoI covalently bound to DNA (lanes 5–8). After the SDS treatment, the samples were treated with proteinase K (Prot K) to confirm the presence of proteins in the retarded bands (lanes 2, 4, 6 and 8). The % of proteins engaged in a complex with dsMCs is indicated; (**b**) Relaxation was performed with the Ct TopoI at a concentration of 18.75 mU μL^−1^ (≈0.2 nM) in the presence of camptothecin (final concentration, 10 μM) as described in the methods section. After 10 minutes of incubation at 37 °C, SDS was added to the reaction mixtures to irreversibly trap the Ct TopoI covalently bound to DNA (lanes 3 and 4). After the SDS treatment, the samples were treated with proteinase K (Prot K) to confirm the presence of proteins in the retarded bands (lanes 2 and 4).

Complexes with Ct TopoI covalently linked to DNA could be detected on an agarose gel under native conditions (Fig. 1a, lane 7). The efficiency of the SDS treatment to trap the protein covalently linked to dsMCs was estimated by comparing the percentage of protein-DNA complexes formed in the presence or absence of SDS. The results indicate that SDS could convert nearly 40% of the Ct TopoI bound to DNA into a covalent complex (Fig. 1a, compare lanes 3 and 7).

**Figure 2:**
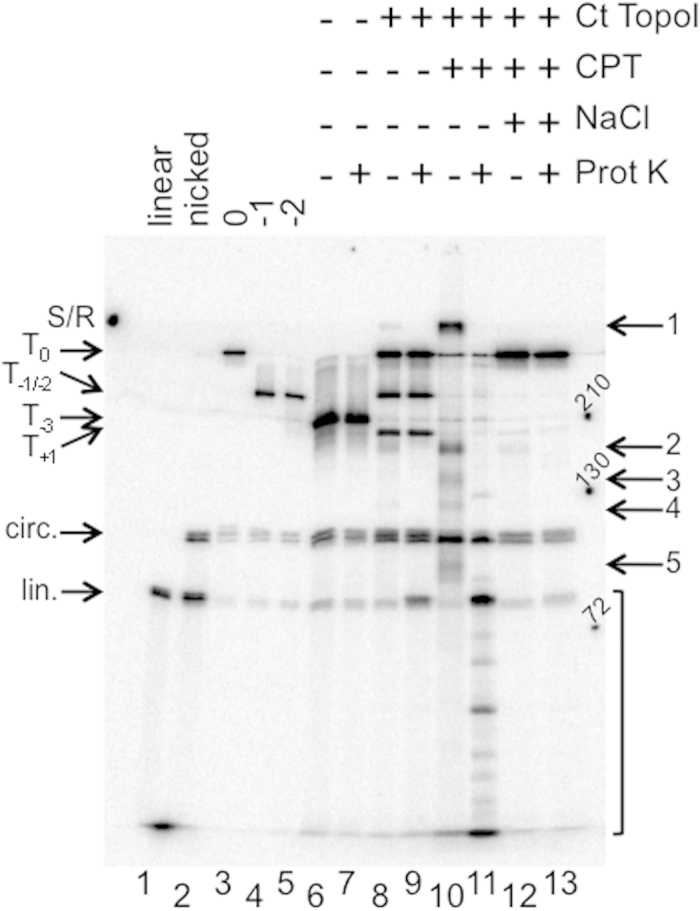
Visualization of the covalent Ct TopoI-DNA complex. Relaxation assay analyzed by SDS-PAGE. The relaxation of topoisomer T_−3_ by the Ct TopoI (at a final concentration, 18.75 mU μL^−1^ (≈0.2 nM)) was performed in the absence (lanes 8 and 9) or in the presence of camptothecin (CPT) at a final concentration of 10 μM (lanes 10–13) as described in the methods section. After 10 minutes of incubation at 37 °C, SDS was added to the reaction mixtures to irreversibly trap the Ct TopoI covalently bound to DNA (lanes 8–13). When indicated, the samples were next treated with proteinase K (Prot K) (lanes 9, 11 and 13). Lanes 12 and 13, NaCl was added to the samples prior to SDS and proteinase K treatments. Lane 1, linear 235 nt-long fragment; lane 2, denatured nicked dsMCs; lane 3, topoisomer T_0_; lane 4, topoisomer T_−1_; lane 5, topoisomer T_−2_; lanes 6 and 7, samples were treated as in lanes 8 and 9, respectively, except that no enzyme was added. S/R, limit between the resolving and the stacking gel. lin., linear. cir., circular. Arrows 1, 2, 3, 4 and 5 on the right side of the gel point to proteinase K-sensitive material. The bracket on the right side of the gel represents DNA species that migrate faster than the linear 235 nt-long fragment. Numbers 210, 130 and 72 on the right side of the gel correspond to the molecular mass of proteins of 210, 130 and 72 kDa, respectively.

As the agarose gel did not resolve the different protein-free DNA species (topoisomers and nicked dsMCs), the reaction products were analyzed on polyacrylamide gels. In theory, the proteinase K treatment should make the radiolabeled species corresponding to the covalent complex disappear. A concomitant enrichment of either (i) linear 235 nucleotide (nt)-long DNA if the sample is fully denatured before electrophoresis, or (ii) nicked plasmid if the sample is not denatured, should be measured. SDS-PAGE revealed radiolabeled material that was sensitive to proteinase K treatment (Fig. 2, compare lanes 8 and 9, arrows 1–2) and a noticeable enrichment of linear DNA upon proteinase K treatment (Fig. 2, compare lanes 8 and 9, arrow labeled “lin.”). Among the two radiolabeled bands sensitive to proteinase K treatment, one of them had an apparent molecular mass close to that of the Ct TopoI-DNA covalent complex (Fig. 2, lane 8, arrow 2, ≈170 kDa). The other species (Fig. 2, lane 8, arrow 1) was trapped at the interface between the resolving and the stacking gel (S/R). When samples were analyzed on chloroquine-containing polyacrylamide gels under native (Supplementary Fig. 4a) or denaturing (Supplementary Fig. 4b) conditions, a similar enrichment of nicked DNA (Supplementary Fig. 4a, compare lanes 7 and 8) or linear 235 nt-long DNA (Supplementary Fig. 4b, compare lanes 7 and 8) was observed. Under such gel conditions, the high molecular weight species that was sensitive to proteinase K treatment (Supplementary Fig. 4a, b, arrow 1) could not penetrate into the gel. The results of these experiments demonstrate that a small amount of the Ct TopoI covalently linked to DNA could be detected during the relaxation of dsMCs.

### Enrichment in the covalent complex in the presence of camptothecin

To confirm that the covalent complex isolated during the relaxation of dsMCs was indeed the covalent complex involved in the reaction cycle of topoisomerases, we characterized the proteinase K-sensitive species in the presence of camptothecin, a topoisomerase drug known to inhibit relaxation by slowing down the rejoining reaction and thus stabilizing the covalent complex^11^,^12^. Trapping of the Ct TopoI in a covalent complex with dsMCs in the presence of camptothecin could be visualized on an agarose gel under native conditions (Fig. 1b). This trapping reaction was highly efficient as all DNA became engaged in a covalent complex (Fig. 1b, lane 3). Furthermore, SDS-PAGE revealed first, that the relaxation was efficiently inhibited by camptothecin as T_0_ and T_+1_ represented less than 10% of the radiolabeled species (Fig. 2, lane 10). Second, among the radiolabeled material sensitive to proteinase K treatment (Fig. 2, compare lanes 10 and 11, arrows 1–5) two of them had the same mobility as those detected in the absence of camptothecin and were clearly enriched by the presence of camptothecin (Fig. 2, lanes 8 and 10, arrows 1 and 2). Third, both linear 235 nt-long DNA and faster migrating species were significantly enriched upon proteinase K treatment (Fig. 2, lane 11, bracket). Fourth, when samples were analyzed on chloroquine-containing polyacrylamide gels, a high molecular weight species sensitive to proteinase K was also detected (Supplementary Fig. 4a, b, lane 9, arrow 1). After proteinase K treatment this species gave rise to either nicked DNA when the sample was not denatured prior to electrophoresis (Supplementary Fig. 4a, lane 10) or DNA ≤235 nts when the sample was denatured (Supplementary Fig. 4b, lane 10, bracket). These results demonstrate that the proteinase K-sensitive material isolated during our experimental conditions corresponds well to the functional covalent topoisomerase-DNA complex.

### Effect of salt on the covalent complex trapped on dsMCs by camptothecin

It is well established that a salt jump can promote DNA religation within the covalent complex before complete dissociation of the protein from the DNA. To establish that the intermediate covalently stabilized on the dsMCs by camptothecin was competent for religation following an increase in salt concentration, relaxation of dsMCs by the Ct TopoI was performed in the presence of camptothecin before successively adding NaCl and finally SDS. The results indicate that the covalent complex stabilized on the dsMCs by camptothecin was indeed competent for religation since T_0_ was the major product of the reaction (Fig. 2, lane 12; Supplementary Fig. 4a, b, lane 11). Its almost complete disappearance agreed well with its capability to complete the reaction cycle.

### Covalent linking of multiple Ct TopoI per strand of dsMC by camptothecin

**Figure 3:**
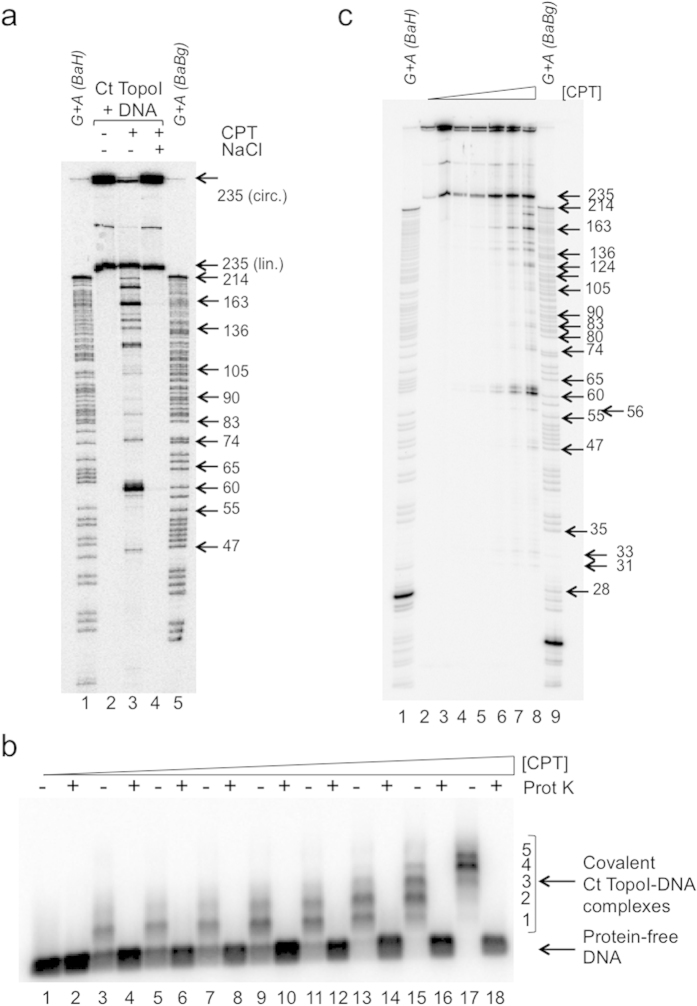
Multiple Ct TopoI can be covalently linked to the dsMCs in the presence of camptothecin. (**a**) The relaxation of a mixture of the topoisomers T_−4_ and T_−5_ by the Ct TopoI (18.75 mU μL^−1^ (≈0.2 nM)) was performed in the absence (lane 2) or in the presence of 10 μM of camptothecin (CPT, lanes 3 and 4). After 10 minutes of incubation at 37 °C, SDS was added to the reaction mixtures to irreversibly trap the Ct TopoI covalently bound to DNA, and the samples were next treated with proteinase K. In lane 4, NaCl was added to the sample prior to SDS and proteinase K treatments. Samples were precipitated and analyzed on a 12% sequencing gel. dsMCs were digested with BamHI and HindIII (lane 1) or with BamHI and BglII (lane 5) and sequenced to determine the positions of the guanines and adenines in the sequence^36^. The size of single-stranded DNA fragments is indicated on the right side of the gel. lin., linear. circ., circular; (**b**) As in (**a**) except for the concentrations of camptothecin (CPT; 0 (lanes 3 and 4); 0.05 (lanes 5 and 6); 0.1 (lanes 7 and 8); 0.25 (lanes 9 and 10); 0.5 (lanes 11 and 12); 1 (lanes 13 and 14); 5 (lanes 15 and 16); 10 μM (lanes 17 and 18)) and the gel analysis (1% agarose gel, native conditions). When indicated, the samples were next treated with proteinase K (Prot K). Protein-free DNA and covalent Ct TopoI-DNA complexes are indicated. Lanes 1 and 2 contained 4% DMSO (no Ct TopoI no camptothecin); (**c**) As in (**a**) except for the concentrations of camptothecin (CPT; 0 (lane 3); 0.1 (lane 4); 0.25 (lane 5); 0.5 (lane 6); 1 (lane 7); 10 μM (lane 8)). dsMCs were digested with BamHI and HindIII (lane 1) or BamHI and BglII (lane 9) and sequenced to determine the positions of the guanines and adenines in the sequence^36^. Lane 2, mixture of T_−4_ and T_−5_. The size of single-stranded DNA fragments is indicated on the right side of the gel.

Electrophoresis on a sequencing gel was used to investigate the size of the material that migrated faster than the 235 nt-long linear DNA fragment when relaxation was performed in the presence of camptothecin (Fig. 2, lane 11, bracket; Supplementary Fig. 4b, lane 10, bracket). This analysis revealed that DNA fragments of ≈45 to 235 nts were produced (Fig. 3a, lane 3). Such fragments might originate from the covalent binding of several Ct TopoI molecules per strand of dsMC. To test this hypothesis, we titrated camptothecin during a relaxation reaction. Analysis on an agarose gel under native conditions demonstrated that up to five distinct complexes with Ct TopoI covalently bound to DNA could be resolved (Fig. 3b), confirming the possibility of covalently linking multiple topoisomerases per dsMC. The increase of the level of incision of the dsMCs as the concentration of the camptothecin increased (Fig. 3c) was also consistent with this possibility. The result that more than one Ct TopoI could be covalently attached per dsMC explains the formation of covalent complexes with an apparent molecular mass smaller than the predicted molecular mass of this complex formed with a 235 nt-long DNA fragment (Fig. 2, arrows 3–5).

### Specificity of the pattern of the camptothecin-induced incision of the dsMCs

**Figure 4:**
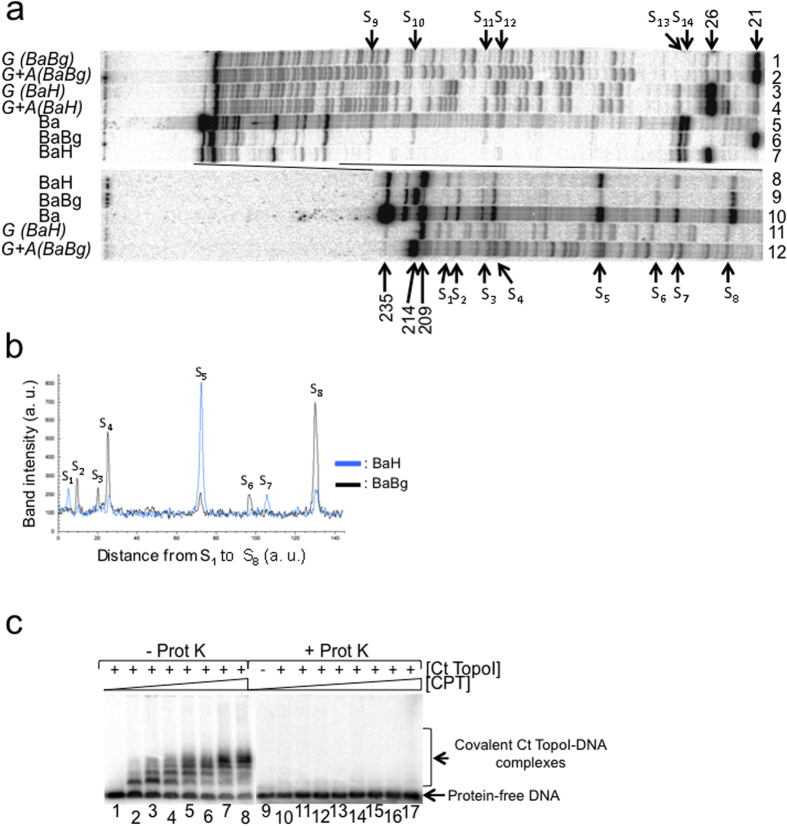
Determination of the sequence of the camptothecin-induced cleavage sites. (**a**) Identification of the camptothecin-induced cleavage sites on the BamHI-digested dsMCs. The dsMC linearized by BamHI was treated with Ct TopoI (18.75 mU μL^−1^ (≈0.2 nM)) in the presence of 10 μM camptothecin as described in the methods section. After 10 minutes of incubation at 37 °C SDS was added to irreversibly trap the Ct TopoI covalently bound to DNA. The sample was next treated with proteinase K and precipitated. Lanes 5 and 10, sample directly loaded on a sequencing gel after precipitation. Sample further digested with BglII (lanes 6 and 9) or HindIII (lanes 7 and 8). dsMCs were digested with BamHI and BglII (lanes 1, 2 and 12) or BamHI and HindIII (lanes 3, 4 and 11) and sequenced to determine the positions of the guanines (lanes 1, 3 and 11) or the guanines and adenines (lanes 2, 4 and 12) in the sequence^36^. Samples loaded onto a 10% (lanes 1–7) or 7% (lanes 8–12) acrylamide sequencing gel. The camptothecin-induced cleavage sites are labelled from S_1_ to S_14_; (**b**) Overlayed scans of lanes 8 and 9 from site S_1_ to S_8_. Black line, scan of lane 8 (BamHI-BglII double digestion). Blue line, scan of lane 9 (BamHI-HindIII double digestion); a. u., arbitrary unit; (**c**) The linearized dsMCs were incubated with Ct TopoI (18.75 mU μL^−1^ (≈0.2 nM)) and increasing concentrations of camptothecin [CPT; 0.05 (lanes 2 and 11); 0.1 (lanes 3 and 12); 0.2 (lanes 4 and 13); 0.5 (lanes 5 and 14); 1 (lanes 6 and 15); 5 (lanes 7 and 16); 10 μM (lanes 8 and 17)] as described in the methods section. After 10 minutes of incubation at 37 °C, SDS was added to the reaction mixtures to irreversibly trap the Ct TopoI covalently bound to DNA. When indicated, the samples were next treated with proteinase K (Prot K). The products of the reactions were finally analyzed on a 1% agarose gel under native conditions. Lane 9 contained 4% DMSO (no Ct TopoI no camptothecin). Lanes 1 and 10 contained 4% DMSO instead of camptothecin.

The radiolabeled nature of the dsMCs made it possible to identify the DNA sequences where the Ct TopoI had established a covalent bond with the DNA during relaxation in the presence of camptothecin. As both strands of the dsMCs were radiolabeled, after the relaxation reaction, the reaction products needed to be submitted to a double restriction enzyme digestion (BamHI + BglII or BamHI + HindIII) to visualize the nicks on one of the two strands (Supplementary Fig. 5). Furthermore, to unambiguously identify the strand that was incised, the pattern of incision obtained after the double digestion was compared with that obtained after the single BamHI digestion. The resistance or sensitivity of a specific band to digestion with the second enzyme (BglII or HindIII) indeed indicated the strand that did or did not carry the nick (Supplementary Fig. 5). Once the strand that carried the nick had been identified, the site of incision was positioned on the sequence by comparison with the sequencing lanes. We first analysed the incision profile of the Ct TopoI in the presence of camptothecin with the BamHI-linearized form of dsMCs. 4 bands along the BamHI digestion profile (Fig. 4a, lanes 5 and 10) were resistant to the additional HindIII digestion and sensitive to the additional BglII digestion (Fig. 4a, compare lanes (5–7), and (8–10); scans comparing intensity of the bands S_1_, S_5_, and S_7_ of lanes 8 and 9), indicating that these 4 incision sites were located on strand 1 of the DNA (Supplementary Fig. 5). Conversely, 8 bands along the BamHI digestion profile (Fig. 4a, lanes 5 and 10) were resistant to the additional BglII digestion and sensitive to the additional HindIII digestion (Fig. 4a, compare lanes (5–7), and (8–10); scans comparing intensity of bands S_2_, S_3_, S_4_, S_6_ and S_8_ of lanes 8 and 9). These 8 incision sites were thus located on strand 2 of the DNA (Supplementary Fig. 5). The disappearance of these 8 bands by HindIII digestion was not complete due to the partial HindIII enzyme digestion. However a difference in intensity of these bands between the BamHI-BglII and BamHI-HindIII double digestions was clearly measurable (Fig. 4b). The bands S_13_ and S_14_ were resistant to BglII and HindIII (Fig. 4a). As the “5′-TTC” sequence that partially overlapped the HindIII restriction site (“5′-AAGCTT”) represented a potential attachment site of the Ct TopoI^23^, it is possible that the nick created by covalent binding of Ct TopoI inhibits the restriction enzyme reaction, thus making these two bands resistant to HindIII. Overall, our results indicate that in the presence of camptothecin, Ct TopoI could covalently bind the 235 bp-long DNA at 12 different positions with 8 incision sites located on strand 2, and 4 on strand 1 of the linear DNA (Fig. 4a,b; Supplementary Fig. 5). The three strong cleavage sites (sites S_4_, S_5_ and S_8_) matched quite well the published consensus sequence^23^ since (i) two of them had a thymine at position −1 (sites S_4_ and S_5_), (ii) all three sites had a guanine at position +1 and (iii) neither a guanine and nor a thymine was found at positions −4 and −3, respectively (Table 1).

The patterns of incision of the topoisomers T_0_, T_−3_ and T_−6_ (shown only for T_−3_) were similar to that of the BamHI-linearized dsMC, and the 12 cleavage sites observed with the linear form of the dsMCs were detectable with the dsMCs (Supplementary Fig. 6, only S_1_ to S_9_ cleavage sites indicated by * are shown). This result is consistent with the observation that the profile of appearance of the covalent complexes trapped by SDS on linear DNA as a function of camptothecin (Fig. 4c) was very similar to that obtained with a mixture of topoisomers T_−4_ and T_−5_ (Fig. 3b). However the dsMC cleavage profiles either singly digested by BamHI (Supplementary Fig. 6, lane 5) or doubly digested by BamHI-BglII (Supplementary Fig. 6, lane 4) or BamHI-HindIII (Supplementary Fig. 6, lane 3) were more complex than their equivalents obtained with the BamHI-linearized dsMCs (Fig. 4a, lanes 7–9). This higher complexity stems from the partial digestion of BamHI giving rise to additional bands detectable on the incision profile of (i) the undigested dsMC (Supplementary Fig. 6, indicated by ⇑ on lanes 5 and 8), (ii) the singly BglII-digested dsMC (Supplementary Fig. 6, indicated by ∇ on lanes 4 and 6) and (iii) the singly HindIII-digested dsMC (Supplementary Fig. 6, indicated by ▲ on lanes 1 and 3). The results of these experiments demonstrate that in the presence of camptothecin, Ct TopoI incised the dsMCs in a linking number independent manner and that the circular nature of the dsMCs did not influence the positioning of the Ct TopoI on the DNA. Therefore the use of the linearized form of dsMCs to determine the consensus sequence of incision of a novel type IB topoisomerase is more straightforward.

### Visualization of the covalent complex during the relaxation of dsMCs by the Ec TopoI

**Figure 5:**
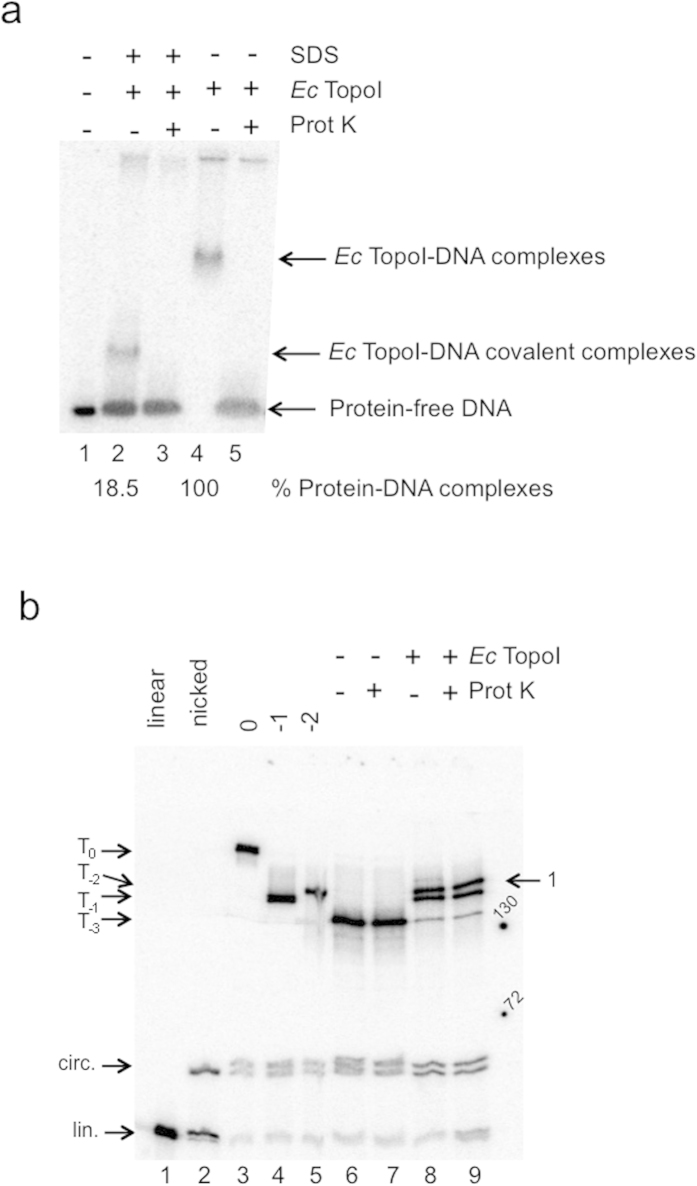
Visualization of the covalent *Ec* TopoI-DNA complex. (**a**) Relaxation assay analysed on an agarose gel under native conditions. Relaxation of topoisomers T_−3_ was performed with the *Ec* TopoI at a concentration of 500 mU μL^−1^ (≈30 nM) (lanes 2–5) as described in the methods section. After an incubation of 10 minutes at 37 °C, SDS was added in the reaction mixtures (lanes 2 and 3) to irreversibly trap the *Ec* TopoI covalently bound to DNA. The samples were next treated with proteinase K (Prot K) to confirm the presence of proteins in the retarded bands (lanes 3 and 5). The % of proteins engaged in a complex with dsMCs is indicated. Protein-free DNA and complexes with *Ec* TopoI covalently linked or not to the DNA are indicated; (**b**) Relaxation assay analyzed by SDS-PAGE. The relaxation of topoisomer T_−3_ was performed by the *Ec* TopoI at a concentration of 500 mM μL^−1^ (≈30 nM) as described in the methods section. After 10 minutes at 37 °C, SDS was added to the reaction mixtures to irreversibly trap the *Ec* TopoI covalently bound to DNA (lane 8). Lane 9, the sample was next treated with proteinase K (Prot K) to detect radiolabeled bands that were sensitive to the proteinase K treatment. Lane 1, linear 235 nt-long fragment; lane 2, denatured nicked dsMCs; lane 3, topoisomer T_0_; lane 4, topoisomer T_−1_; lane 5, topoisomer T_−2_; lanes 6 and 7, samples were treated as in lanes 8 and 9, respectively, except that no enzyme was added. Arrow 1 on the right side of the gel points to the proteinase K-sensitive material. Numbers 130 and 72 on the right side of the gel correspond to the molecular mass of proteins of 130 and 72 kDa, respectively. lin., linear. cir., circular.

Our method made it possible to visualize the covalent complex Ct TopoI-DNA during the relaxation of dsMCs. We wondered whether the covalent complex could also be detected with another topoisomerase, the *Ec* TopoI, since this topoisomerase was able to efficiently relax most dsMCs (Supplementary Figs. 2b, 3b). Complexes of *Ec* TopoI covalently linked to DNA could be detected on an agarose gel under native conditions (Fig. 5a, lane 2). Whereas SDS could convert nearly 40% of the Ct TopoI bound to DNA into a covalent complex (Fig. 1a, compare lanes 3 and 7), less that 20% of the *Ec* TopoI bound to DNA could be irreversibly linked to DNA by the SDS treatment (Fig. 5a, compare lanes 2 and 4), indicating that the efficiency of the SDS treatment was topoisomerase-dependent.

A covalent *Ec* TopoI-DNA complex could also be detected by SDS-PAGE as a proteinase K-sensitive species (Fig. 5b, arrow 1) and a slight enrichment of linear DNA were clearly observed (Fig. 5b, compare lanes 8 and 9). When samples were analyzed on chloroquine-containing polyacrylamide gels under native (Supplementary Fig. 7a) or denaturing (Supplementary Fig. 7b) conditions, a high molecular weight species (Supplementary Fig. 7a, b, lane 7, arrow 1) that failed to enter into the gel and that was sensitive to proteinase K treatment (Supplementary Fig. 7a, b, compare lanes 7 and 8) was detected. As with Ct TopoI, a small amount of *Ec* TopoI was covalently bound to DNA during the relaxation of dsMCs. In agreement with the results obtained by the analysis on an agarose gel, a smaller proportion of *Ec* TopoI than of Ct TopoI was covalently trapped on DNA.

## Discussion

DsMCs have been described as powerful substrates to investigate the structure of specific DNA sequences since the influence of adjacent random sequences is minimized^24^. They have also been widely used in the past by Prunell’s laboratory to investigate multiple aspects of the chromatin assembly process^25^,^26^,^27^,^28^,^29^,^30^. In addition to allowing to measure the change of linking number per relaxation step of the topoisomerase reaction cycle^31^ and specific kinetic and partitioning parameters of relaxation^32^, they offer the possibility to dissect other key biochemical features of the relaxation reactions performed by topoisomerases. As reported here, the quantitative analysis of relaxation reactions in terms of the proportion of each topoisomer as a function of enzyme concentration is indeed made possible by the radioactive nature of the DNA substrates and the possibility of resolving the topoisomers by one dimensional PAGE (Supplementary Fig. 1b). We validated the use of radiolabeled dsMCs of variable linking numbers to characterize topoisomerases by first testing the relaxation activity of two topoisomerase models, the *Ec* TopoI and the Ct TopoI, on these different topoisomers (Supplementary Figs. 2, 3). Both topoisomerases indeed relaxed the dsMCs efficiently, although with differences not only in the reaction yield but also in the final product. The *Ec* TopoI was unable to fully relax the dsMCs since the major final product was T_−1_, a result which is in agreement with the early observation made by Wang in 1971 that the relaxation of negatively supercoiled plasmid does not go to completion, probably because of the slow rate of the reaction when the degree of superhelicity is low^33^. In contrast, the Ct TopoI fully relaxed all substrates.

Second, using a method that relied on the use of radiolabeled supercoiled dsMCs and a combination of gels to analyze the products of a relaxation reaction (agarose gels, polyacrylamide gels with SDS or chloroquine), we succeeded in visualizing the covalent topoisomerase-DNA intermediate during a relaxation reaction (Figs. 1, 2, 3, 5, Supplementary Figs. 4, 7). The covalent topoisomerase-DNA complex is a transient species of the relaxation reaction with a very short half-life time. As a consequence, its detection and isolation during a relaxation reaction has been very difficult. Our method has filled this gap since the proportion of covalent complexes formed during a relaxation reaction and trapped by SDS has been estimated for both topoisomerases (≈40% and ≈20% for the Ct TopoI and *Ec* TopoI, respectively; Figs. 1a, 5a). Its dependence with respect to the topoisomerase possibly reflects mechanistic differences between the type IA and type IB families of topoisomerases, especially in the mechanism conferring processivity. The molecular mass of the covalent complex measured on the SDS-PAGE, although close to the expected molecular mass in the case of the Ct TopoI covalent complex (165 kDa *versus* ≈ 170 kDa for the expected *versus* the measured molecular mass), greatly differs from the expected molecular mass in the case of the *Ec* TopoI covalent complex (170 kDa *versus* ≈ 260 kDa for the expected *versus* the measured molecular mass). The difference between expected and apparent molecular mass might stem from the difference in the nature of the species that are monitored (protein for the protein molecular weight marker and protein-DNA species for our samples). Discrepancies between predicted and observed buoyant densities have been reported in the case of the protein-DNA covalent complex formed with the DNA untwisting enzyme^13^.

Third, as expected, the covalent Ct TopoI-DNA intermediate was greatly enriched when the relaxation was performed in the presence of camptothecin (Figs. 1, 2, 3, Supplementary Fig. 4), consistent with the covalent linking of multiple topoisomerases per dsMC (Fig. 3). This species was competent for religation following an increase of salt (Fig. 2, Supplementary Fig. 4), and interestingly, T_0_ was the major product of the reaction under these experimental conditions. Other products (such as T_+1_ and T_−1_, clearly detectable during a relaxation reaction without camptothecin) were surprisingly barely detectable, suggesting that camptothecin targets the covalent complex trapped with relaxed DNA but cannot insert into the DNA of the covalent Ct TopoI-DNA complex assembled on the topoisomers T_+1_ or T_−1_.

Fourth, our analysis of the incision profiles as a function of supercoiling revealed that in the presence of camptothecin, Ct TopoI established covalent bonds at positions on dsMCs that were influenced neither by the linking number nor by the circular nature of the substrate (Fig. 4, Supplementary Figs. 5, 6). Although the number of incision sites is small (12), the sequence of the three strongest sites agrees quite well with the published consensus sequence^23^ with thymine at position −1 and guanine at position +1 in 73.5% and 100% of the cases, respectively (Fig. 4 and Table 1). The frequency of detectable cleavage sites ranged from ≈1/30 to ≈1/60 for the strand that carries 8 and 4 sites, respectively. This percentage is low compared to the frequency published previously^23^ and may stem from the reaction conditions that differ between the studies.

All together our data show that radiolabeled dsMCs constitute a powerful alternative substrate to plasmids and oligonucleotides to biochemically characterize the relaxation mechanism of any new topoisomerase, either wild type or mutant, alone or with its binding partners. In addition, our method makes it possible to detect the covalent complex with the preferred and natural substrate of the topoisomerase-catalyzed relaxation reaction. It therefore constitutes a novelty and an improvement in the field of topoisomerases since most of the *in vitro* experiments investigating the nicking-joining reaction and the mechanism of action of topoisomerase drugs use DNA substrates (such as oligonucleotides of various configurations; ss; ds-ss junctions *etc*) that do not position the topoisomerase in its functional state *e.g.* ready to change the topology. Consequently, relationships between the proportion of covalent complex trapped and the efficiency of relaxation will possibly be established in the future. Finally, our study demonstrates that dsMCs are powerful tools to investigate the mechanism of action of topoisomerase drugs during a relaxation reaction, especially molecules that act by stabilizing the covalent topoisomerase-DNA complex.

## Methods

### Material

The calf thymus DNA topoisomerase I (Ct TopoI) was from Invitrogen. The *Escherichia coli* Topoisomerase I (*Ec* TopoI), T4 polynucleotide kinase, calf intestinal phosphatase, T4 DNA ligase, High Fidelity BamHI (HF-BamHI), BglII, High Fidelity HindIII (HF-HindIII) Nb., and BbvCI were from New England Biolabs. [γ-^32^P]-ATP was from Perkin Elmer. The PageRuler Plus prestained protein ladder was from Thermoscientific. The plasmid pHCO3 has been described elsewhere^34^.

### Preparation of ds DNA mini-circles (dsMCs) with a specific relative linking number

To prepare dsMCs of 235 bps, the pHCO3 plasmid was digested by HF-BamHI [1U μL^−1^, 4 h, 37 °C in CutSmart buffer (50 mM KOAc, 20 mM Tris-OAc, 10 mM Mg(OAc)_2_, pH 7.9)] and dephosphorylated by calf intestinal phosphatase (33 mU μL^−1^, 2 h, 37 °C in CutSmart buffer), leading to DNA fragments of 235 and 2765 bps. The calf intestinal phosphatase was inactivated by phenol, phenol:chloroform and chloroform extraction. The dephosphorylated DNA fragment of 235 bps was gel-purified, radiolabeled with polynucleotide kinase [2 μM [γ-^32^P]-ATP, 0.6 U μL^−1^, 30 min, 37 °C in polynucleotide kinase buffer (70 mM Tris-HCl, 10 mM MgCl_2_, 5 mM DTT, pH 7.6)] and ligated with T4 DNA ligase (2 U μL^−1^, 4 h, 16 °C in T4 DNA ligase buffer (50 mM Tris-HCl, 10 mM MgCl_2_, 1 mM ATP, 10 mM DTT, pH 7.5) in the presence of various concentrations of ethidium bromide (from 0 to 20 mg mL^−1^). After ethidium bromide extraction, the covalently closed dsMCs were gel-purified on a 4% acrylamide (29:1 = acryalamide:bisacrylamide mass ratio) gel without chloroquine and recovered by electro-elution (Supplementary Fig. 1a). A specific relative linking number has been attributed to each topoisomer using the band counting method^35^. The relative linking number of 0 has been arbitrarily attributed to the dsMC obtained by ligation without ethidium bromide. When starting with the purified linear 235 bp-long fragment, the yield of preparation of dsMCs ranges from 25% to 2%.

### Relaxation assay

Unless indicated otherwise, the relaxation assays were performed as follows and in 20 μL of Ct buffer (10 mM Tris-HCl, 26 mM KCl, 8 mM MgCl_2_, 500 μM DTT, pH 7.5) for the Ct TopoI or 20 μL of *Ec* buffer (20 mM Tris-OAc, 50 mM KOAc, 10 mM Mg(OAc)_2_, 1 mM DTT, pH 7.9) for the *Ec* TopoI. 1 nM of dsMCs of indicated topology or of the 235 bp-long fragment linearized by BamHI were pre-incubated for 5 min at 37 °C in relaxation buffer before adding the enzyme at the indicated concentration. After 10 min of incubation at 37 °C, the relaxation reactions were stopped by adding SDS (final concentration, 1%) to trap some or all of the topoisomerases covalently linked to DNA. The samples were next treated with proteinase K (final concentration, 1.2 mg mL^−1^; 10 min at 37 °C followed by 10 min at 56 °C) and precipitated with ethanol. When used, camptothecin was diluted in DMSO, added to a final concentration of up to 10 μM (leading to 4% DMSO in the reaction mixture) and pre-incubated with the DNA for 5 min at 37 °C prior to enzyme addition. When NaCl was used to promote DNA religation within the covalent complex, its final concentration was 0.5 M and it was added at the end of the incubation period between the topoisomerase and the dsMCs.

### Conditions of gel analysis

All gels were made with TBE 0.5x (44.5 mM Tris-Base, 44.5 mM boric acid, 1 mM EDTA). When the samples were analyzed under native conditions on an agarose gel, at the end of the relaxation reaction the samples were not precipitated and glycerol was added to a concentration of 4% before loading of the samples onto the 1% agarose gel.

When the samples were analyzed under native conditions on a polyacrylamide gel, after ethanol precipitation the pellets were redissolved in TE supplemented with 4% glycerol and loaded onto a 6% polyacrylamide (29:1 = acrylamide:bisacrylamide mass ratio) gel supplemented with chloroquine (final concentration, 30 μg mL^−1^).

When the samples were analyzed under denaturing conditions on a polyacrylamide gel, after ethanol precipitation the pellets were redissolved in a denaturing loading buffer (TE supplemented with 4% glycerol and 0.1 M NaOH) before being analyzed on a 6% polyacrylamide (29:1 = acrylamide: bisacrylamide mass ratio) gel supplemented with chloroquine (final concentration, 30 μg mL^−1^). [Note that to prevent the loss of the ss DNA (either circular or linear) during electrophoresis and to maintain a good resolution of the topoisomers, the 6% polyacrylamide gel (38 mL) was poured above a 12% polyacrylamide gel (12 mL; 29:1 = acrylamide:bisacrylamide mass ratio)].

When the samples were analyzed by SDS-PAGE, at the end of the relaxation reaction the samples were not precipitated but denatured with SDS (final concentration, 3.25%), formamide (final concentration, 3%) and heat (5 min, 95 °C); their pH was adjusted to 6.8. [Note that the reticulation of the stacking part of the protein gel was 80:1 (acrylamide: bisacrylamide mass ratio)].

### Conditions of digestion with restriction and nicking enzymes

The digestion of dsMCs by BamHI, BglII and HindIII were performed for 18 h at 37 °C in 200 μL in the buffer recommended by the manufacturer (20 mM Tris-OAc, 50 mM KOAc, 10 mM Mg(OAc)_2_, pH 7.9 for BamHI and HindIII, and 50 mM Tris-HCl, 100 mM NaCl, 10 mM MgCl_2_, pH 7.9 for BglII) with the restriction enzyme at a concentration of 1.5 U μL^−1^. To prepare nicked dsMCs, dsMCs were treated with the Nb.BbvCI nicking enzyme (50 mU μL^−1^, 1 h, 37 °C in 20 mM Tris-OAc, 50 mM KOAc, 10 mM Mg(OAc)_2_, 1 mM DTT, pH 7.9 buffer).

### Sequencing reactions

To sequence the 235 nt-long DNA, the 235 bp-long fragment linearized by BamHI was completely digested by BglII or HindIII according to the manufacturer’s recommendations and sequenced according to the Maxam and Gilbert procedures specific for either guanines or guanines and adenines^36^.

## Additional Information

**How to cite this article**: Millet, A. *et al.* Use of double-stranded DNA mini-circles to characterize the covalent topoisomerase-DNA complex. *Sci. Rep.* **5**, 13154; doi: 10.1038/srep13154 (2015).

## Supplementary Material

Supplementary Information

## Figures and Tables

**Table 1:**
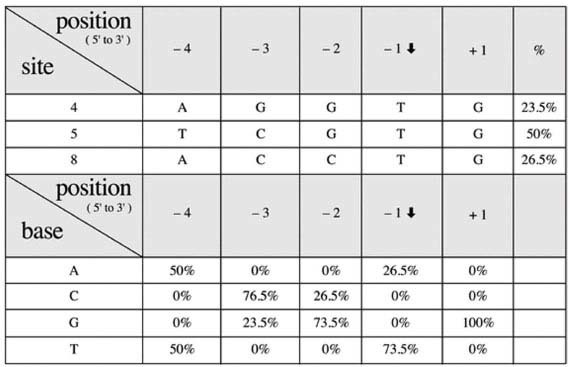
Sequences of the strong cleavage sites and percentage of observed bases at cleavage sites.

The upper part of the table shows the sequence at the designated site. The vertical arrow (_↓_) indicates the cleavage site. The right column (%) represents the relative percentage of incision at the designated site. The lower part of the table represents the percentage of each of the four bases taking into account the relative percentage of incision at each site.
