# Supplementary material for: Use of double-stranded DNA mini-circles to characterize the covalent topoisomerase-DNA complex
Source: Sci Rep. 2015 Aug 24;5:13154. doi: 10.1038/srep13154 (PMC4547392; doi:10.1038/srep13154)

SUPPLEMENTARY DATA

Use of double-stranded DNA mini-circles to characterize the covalent topoisomerase-DNA complex

Armêl Millet[1], François Strauss[1] and Emmanuelle Delagoutte[1]

[1] Structure et Instabilité des Génomes, Sorbonne Universités, Muséum national d'Histoire naturelle, Inserm U 1154, CNRS UMR 7196; CP26, 57 rue Cuvier 75005 Paris, France

Correspondence should be addressed to E.D. (emmanuelle.delagoutte@mnhn.fr)

FIGURE LEGENDS

Supplementary Figure 1 Procedure of preparation of dsMCs. (**a**) The preparation of dsMCs includes several steps. (1) Radiolabeling of the linear 235 bp-long ds DNA with [γ-^{32}P]-ATP. (2) Ligation at various concentrations of EtBr (from 0 to 20 mg mL^{-1}). (3) EtBr extraction, DNA precipitation. (4) PAGE to separate the different species. (5) Cutting of the bands of the gel corresponding to the dsMCs, DNA electro-elution and precipitation. The dsMCs are eluted from the gel that does not contain chloroquine (left), where the dsMCs, the single-stranded linear and double-stranded linear fragments are indicated. The gel with chloroquine at 30 μg mL^{-1} (right) shows the sequential formation of the topoisomers as a function of [EtBr] present in the ligation step and their relative migration. The timing proposed on the left side of the figure with step (4) taking placing overnight (ON) makes the preparation of dsMCs a two-day procedure; (**b**) Resolution of the 7 topoisomers by electrophoresis on a chloroquine-containing gel. The chloroquine in the gel and running buffer was at 30 μg mL^{-1}. Lane 1 contains topoisomer T_0; lane 2, topoisomer T_{-1}; lane 3, topoisomer T_{-2}; lane 4, topoisomer T_{-3}; lane 5, a mixture of topoisomers T_{-4} and T_{-5}; lane 6, topoisomer T_{-5}; lane 7, topoisomer T_{-6}.

Supplementary Figure 2 Relaxation of the topoisomer T_{-2} by the Ct TopoI and the topoisomer T_{-6} by the *Ec* TopoI. Electrophoresis was performed on a chloroquine-containing gel. The chloroquine in the gel and running buffer was at 30 μg mL^{-1}. (**a**) Relaxation by Ct TopoI. Lane 1, topoisomer T_0; lane 2, topoisomer T_{-1}; lane 3, topoisomer T_{-2}; lanes 4 to 11 contain 37.5, 18.75, 9.38, 4.69, 2.34, 1.17, 0.59, 0.29 mU μL^{-1} of Ct TopoI, respectively. Comparison of protein titration curves made it possible to estimate that 18.75 mU μL^{-1} of Ct TopoI corresponded to ≈ 0.2 nM. (**b**) Relaxation by *Ec* TopoI. Lane 1, topoisomer T_0; lane 2, a mixture of topoisomers T_{-1} and T_{-2}; lane 3, a mixture of topoisomers T_{-3} and T_{-4}; lane 4, topoisomer T_{-5}; lane 5, topoisomer T_{-6}; lanes 6 to 13 contain 0.05, 0.15, 0.46, 1.39, 4.17, 12.5, 37.5, 75 mU μL^{-1} of *Ec* TopoI, respectively. Comparison of protein titration curves made it possible to estimate that 18.75 mU μL^{-1} of *Ec* TopoI corresponded to ≈ 1 nM.

Supplementary Figure 3 Quantification of the relaxation of the dsMCs by the Ct TopoI and the *Ec* TopoI. Comparison of protein titration curves made it possible to estimate that 18.75 mU μL^{-1} of Ct TopoI or *Ec* TopoI corresponded to ≈ 0.2 or ≈ 1 nM, respectively.

(**a**) The percentage of product formed after 10 min as a function of the concentration of Ct TopoI for topoisomer T_0 (black line and ×), topoisomer T_{-1} (black line and ◇), topoisomer T_{-2} (black line and □) and a mixture of topoisomers T_{-4} and T_{-5} (black line and ○); (**b**) The percentage of product formed after 10 min of incubation at 37°C as a function of the concentration of *Ec* TopoI for topoisomer T_0 (black line and ×), topoisomer T_{-1} (black line and ◇), topoisomer T_{-2} (black line and □), a mixture of topoisomers T_{-4} and T_{-5} (black line and ○), topoisomer T_{-5} (black line and ■) and topoisomer T_{-6} (black line and ●). Errors bars correspond to the standard deviation calculated from at least two independent experiments.

Supplementary Figure 4 Visualization of the covalent Ct TopoI-DNA complex. The relaxation of topoisomer T_{-3} by the Ct TopoI (at a final concentration, 18.75 mU μL^{-1} (≈ 0.2 nM)) was performed in the absence (lanes 7 and 8) or in the presence of camptothecin (CPT) at a final concentration of 10 μM (lanes 9-12) as described in the methods section. After 10 minutes of incubation at 37°C, SDS was added to the reaction mixtures to irreversibly trap the Ct TopoI covalently bound to DNA (lanes 7-12). When indicated, the samples were next treated with proteinase K (Prot K) to remove the proteins (lanes 8, 10 and 12). Lanes 11 and 12, NaCl was added to the samples prior to SDS and proteinase K treatments. Lanes 5 and 6, samples were treated as in lanes 7 and 8, respectively, except that no enzyme was added. Arrow 1 on the right side of the gel points to the proteinase K-sensitive material. (**a**) Relaxation assay analyzed on a chloroquine-containing 6% polyacrylamide gel under native conditions. The chloroquine in the gel and running buffer was at 30 $\mu g\ mL^{-1}$. Lane 1, nicked dsMCs; lane 2, topoisomer T_0; lane 3, topoisomer T_{-1}; lane 4, topoisomer T_{-2}; (**b**) As in (a) except that the samples were analyzed under denaturing conditions on a chloroquine-containing 6% polyacrylamide gel. The bracket on the right side of the gel represents DNA species that migrate faster than the linear 235 nt-long fragment. lin., linear. cir., circular.

Supplementary Figure 5 Principle of the analysis of the camptothecin-induced cleavage sites of the dsMCs by the Ct TopoI. The relative position of the BamHI, BglII and HindIII restriction sites is shown and the size of the smallest fragment generated by the double BamHI-BglII and BamHI-HindIII digestions is indicated. The dsMC has a green (strand 1) and a black (strand 2) strand. The radioactive phosphorus is represented by a circle filled in green or black, according to the color of the strand. The nick (indicated by

an arrow) is present either on the green (left) or black (right) strand. To identify the strand that is nicked, the profile of the single BamHI digestion must be compared with that of the double BamHI-BglII and BamHI-HindIII digestions. Left: The nick on the dsMC is located on the green strand. The single BamHI digestion of the dsMCs generates fragments of 235 and n nts. The additional BglII digestion generates a 214 and a 21 nt-long fragment (from the 235 and n nt-long fragments, respectively), whereas the supplementary HindIII digestion leaves the n nt-long fragment intact but shortens the 235 nt-long fragment into a 26 nt-long fragment. Right: The nick on the dsMC is located on the black strand. The single BamHI digestion of the dsMCs generates fragments of 235 and m nts. The supplementary BglII digestion leaves the m nt-long fragment intact but shortens the 235 nt-long fragment into a 21 nt-long fragment, whereas the additional HindIII digestion generates a 209 and a 26 nt-long fragment (from the 235 and m nt-long fragments, respectively).

Supplementary Figure 6 Identification of the camptothecin-induced cleavage sites of the dsMCs by the Ct TopoI. All samples were loaded on a 7% acrylamide sequencing gel. The indicated dsMCs (T_0 or T_{-3}) were incubated with Ct TopoI (final concentration, 18.75 mU μL^{-1} (≈ 0.2 nM)) in the presence of camptothecin (final concentration, 10 μM), and after 10 minutes of incubation at 37°C SDS was added to irreversibly trap the Ct TopoI covalently bound to DNA. The samples were next treated with proteinase K and precipitated. The samples were either loaded directly onto the sequencing gel (lane 8), or singly digested with HindIII (lane 1), BamHI (lane 5) or BglII (lane 6), or doubly digested with BamHI and HindIII (lane 3) or BamHI and BglII (lane 4) before being loaded directly onto the sequencing gel. Lanes 2 and 12, dsMCs were digested with BamHI and HindIII and sequenced according to the Maxam and Gilbert procedure to determine the positions of the guanines and adenines in the sequence (1). Lanes 7 and 11, dsMCs were digested with BamHI and HindIII and sequenced according to the Maxam and Gilbert procedure to determine the positions of the guanines in the sequence (1). Lane 9, dsMCs were digested with BamHI and BglII and sequenced according to the Maxam and Gilbert procedure to determine the positions of the guanines in the sequence (1). Lane 10, dsMCs were digested with BamHI and BglII and sequenced according to the Maxam and Gilbert procedure to determine the positions of the adenines and guanines in the sequence (1). The camptothecin-induced cleavage sites

are designated by a *. ⇧, main material when the reaction products have not been digested by any restriction enzyme. ▽, main material when the reaction products have only been digested by BglII. ▲, main material when the reaction products have only been digested by HindIII. (1) Maxam, A. M. & Gilbert, W. A new method for sequencing DNA. *Proc. Natl. Acad. Sci. U. S. A.* **74,** 560–4 (1977).

Supplementary Figure 7 Visualization of the covalent *Ec* TopoI-DNA complex. The relaxation of topoisomer T_{-3} was performed by the *Ec* TopoI at a concentration of 500 mM μL^{-1} (≈ 30 nM) (lanes 7 and 8). After 10 min at 37°C, SDS was added to the reaction mixtures to irreversibly trap the *Ec* TopoI covalently bound to DNA (lanes 7 and 8). Lane 8, the sample was next treated with proteinase K (Prot K) to identify proteinase K-sensitive radiolabeled material. Lanes 5 and 6, samples were treated as in lanes 7 and 8, respectively, except that no enzyme was added. Lane 1, nicked dsMCs; lane 2, topoisomer T_0; lane 3, topoisomer T_{-1}; lane 4, topoisomer T_{-2}. Arrow 1 on the right side of the gel points to the proteinase K-sensitive material. (**a**) Relaxation assay analyzed under native conditions on a chloroquine-containing 6% polyacrylamide gel. The chloroquine in the gel and in the running buffer was at 30 $\mu g\ mL^{-1}$; (**b**) Relaxation assay analyzed on a polyacrylamide gel under denaturing conditions. As in (a) except that the samples were analyzed under denaturing conditions on a chloroquine-containing 6% polyacrylamide gel. lin., linear. cir., circular.

Supplementary Figure 1 (Delagoutte)

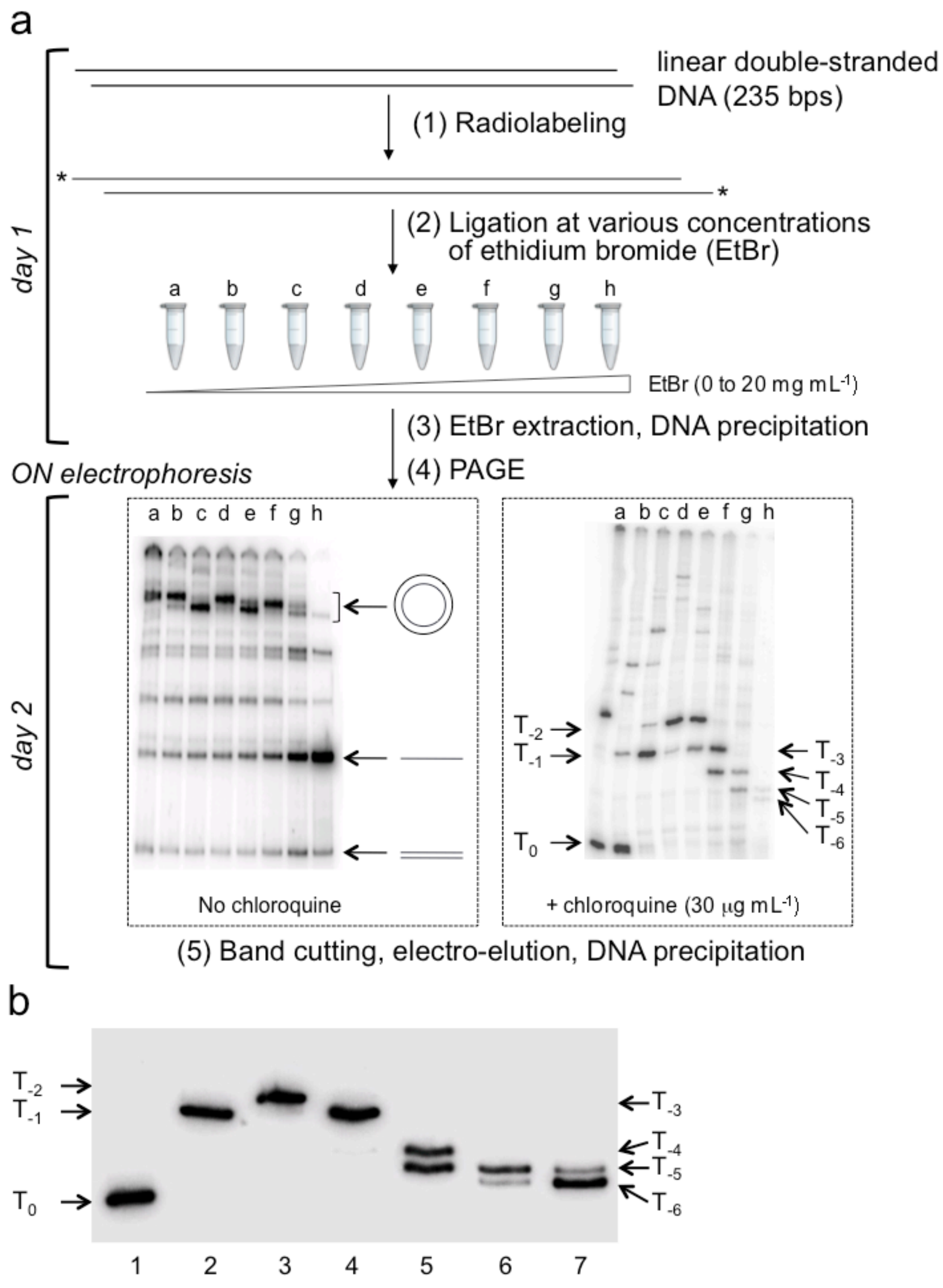

Supplementary Figure 2 (Delagoutte)

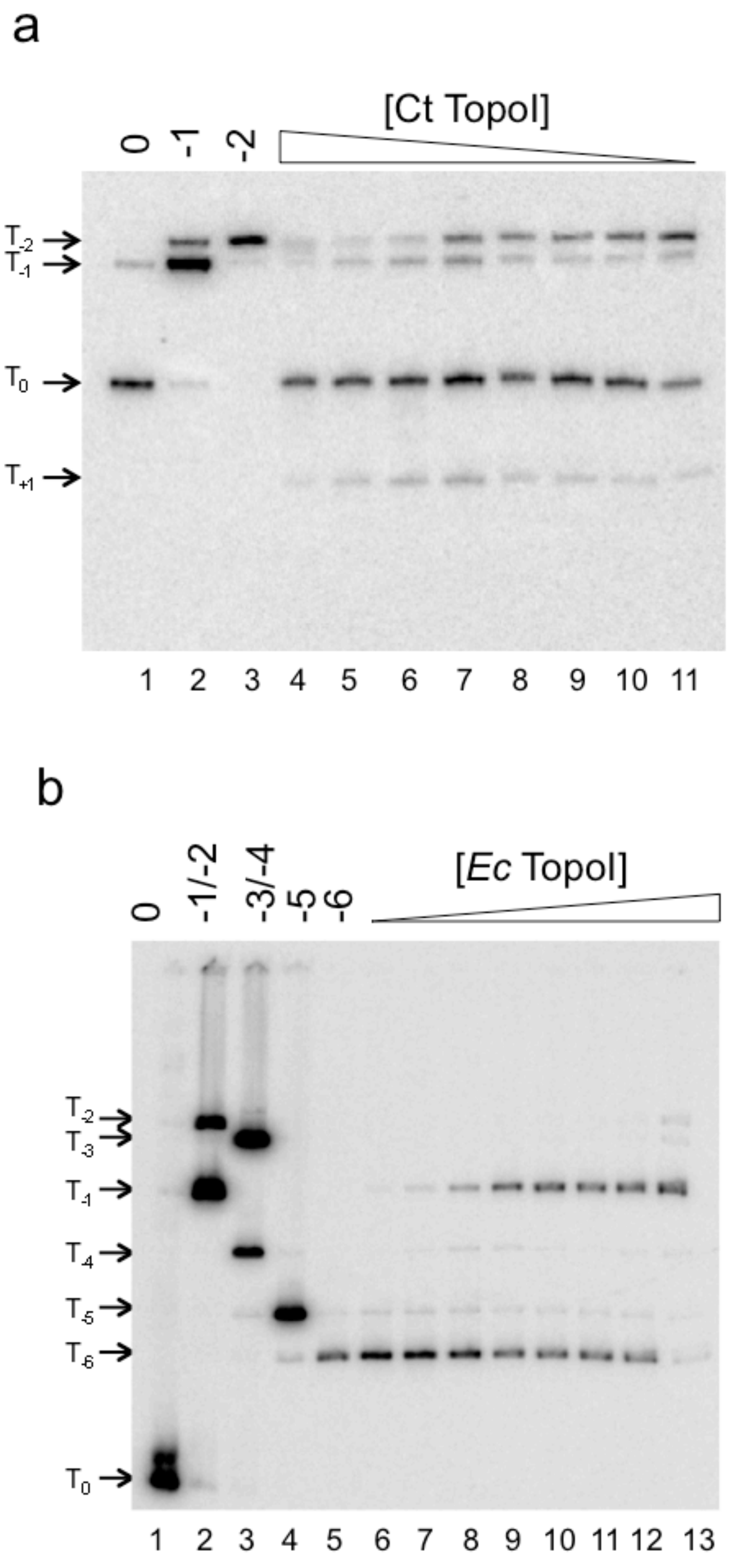

Supplementary Figure 3 (Delagoutte)

a

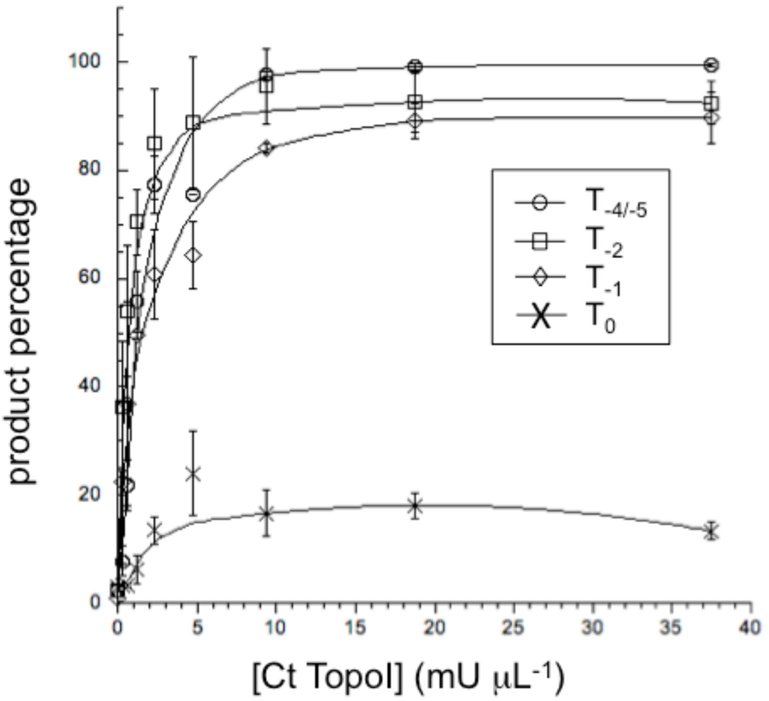

b

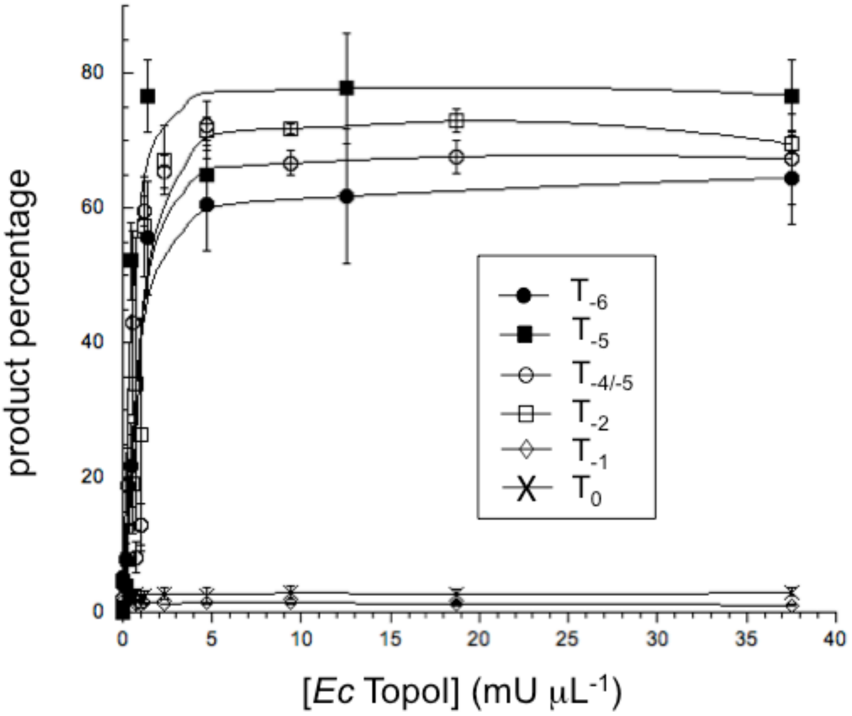

Supplementary Figure 4 (Delagoutte)

a

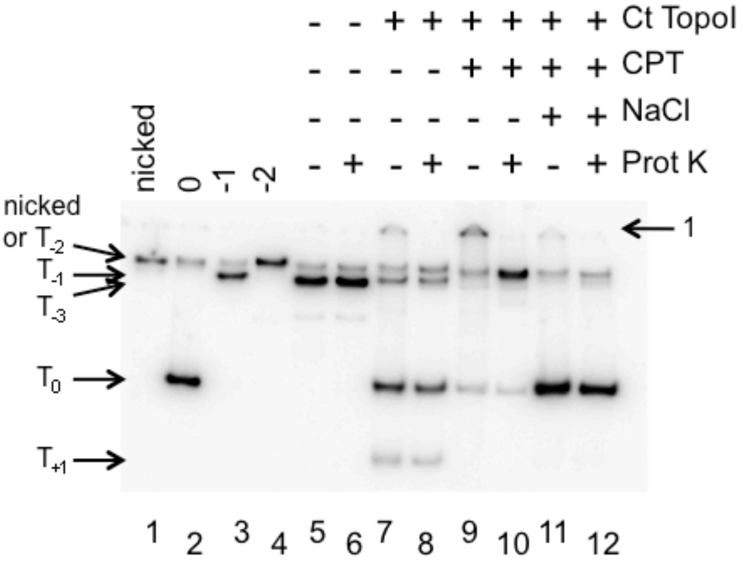

b

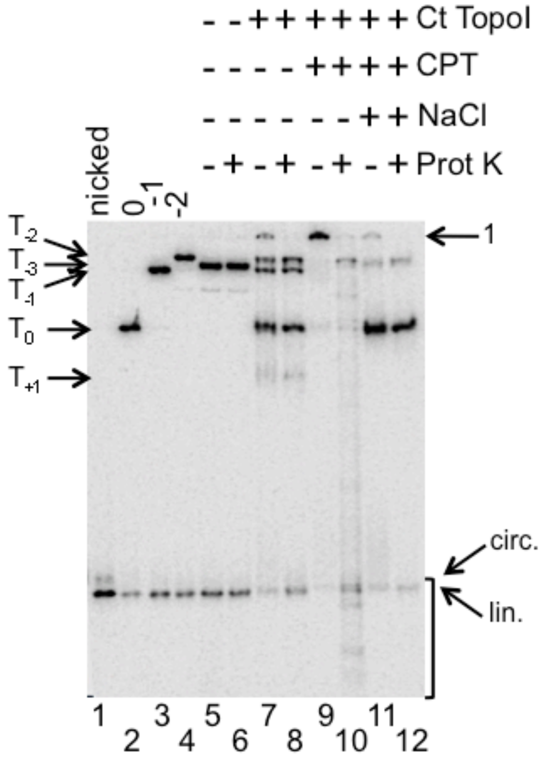

Supplementary Figure 5 (Delagoutte)

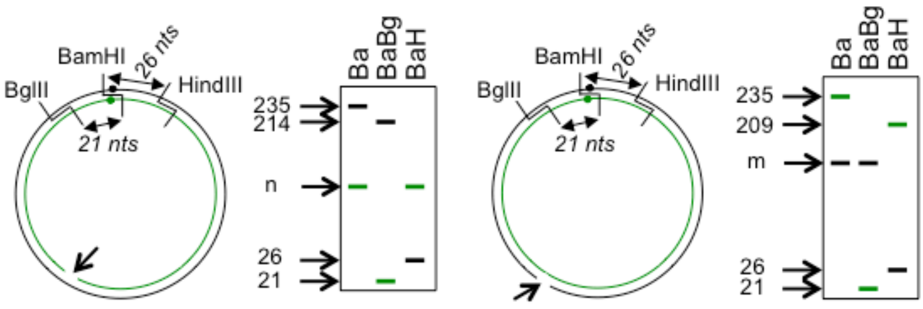

Supplementary Figure 6 (Delagoutte)

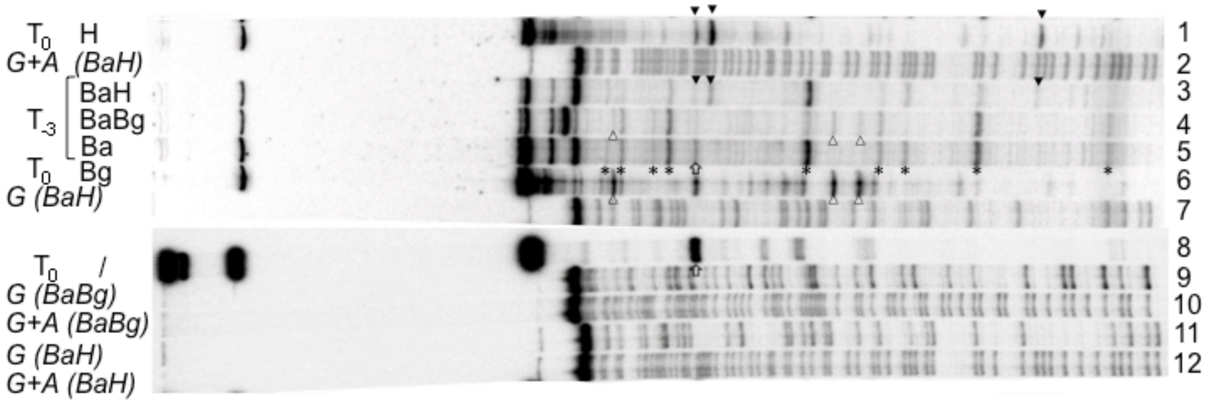

Supplementary Figure 7 (Delagoutte)

a

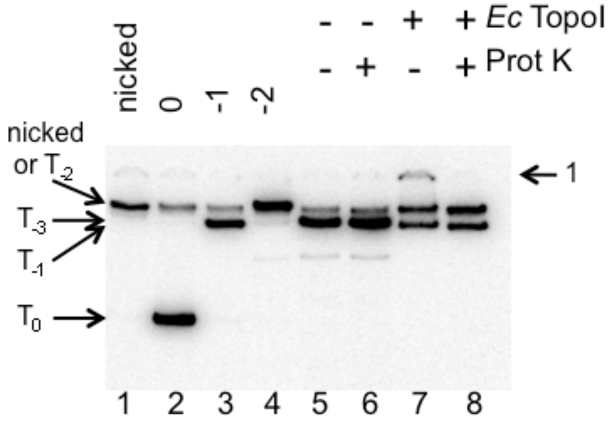

b

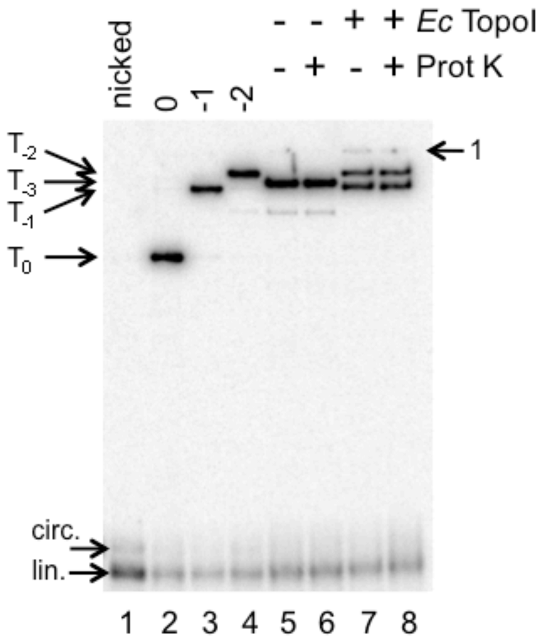

Supplement: Supplementary Information [file srep13154-s1.pdf]
